# Enhancing therapeutic efficacy in triple‐negative breast cancer and melanoma: synergistic effects of modulated electro‐hyperthermia (mEHT) with NSAIDs especially COX‐2 inhibition in *in vivo* models

**DOI:** 10.1002/1878-0261.13585

**Published:** 2024-01-12

**Authors:** Nino Giunashvili, Jeremiah Mbuotidem Thomas, Csaba András Schvarcz, Pedro Henrique Leroy Viana, Kenan Aloss, Syeda Mahak Zahra Bokhari, Zoltán Koós, Dániel Bócsi, Enikő Major, Andrea Balogh, Zoltán Benyó, Péter Hamar

**Affiliations:** ^1^ Institute of Translational Medicine, Semmelweis University Budapest Hungary; ^2^ HUN‐REN‐SU Cerebrovascular and Neurocognitive Diseases Research Group Budapest Hungary

**Keywords:** COX‐2 antagonist, melanoma, modulated electro‐hyperthermia, NSAID, triple‐negative breast cancer

## Abstract

Triple‐negative breast cancer (TNBC) is a leading cause of cancer mortality and lacks modern therapy options. Modulated electro‐hyperthermia (mEHT) is an adjuvant therapy with demonstrated clinical efficacy for the treatment of various cancer types. In this study, we report that mEHT monotherapy stimulated interleukin‐1 beta (IL‐1β) and interleukin‐6 (IL‐6) expression, and consequently cyclooxygenase 2 (COX‐2), which may favor a cancer‐promoting tumor microenvironment. Thus, we combined mEHT with nonsteroid anti‐inflammatory drugs (NSAIDs): a nonselective aspirin, or the selective COX‐2 inhibitor SC236, *in vivo*. We demonstrate that NSAIDs synergistically increased the effect of mEHT in the 4T1 TNBC model. Moreover, the strongest tumor destruction ratio was observed in the combination SC236 + mEHT groups. Tumor damage was accompanied by a significant increase in cleaved caspase‐3, suggesting that apoptosis played an important role. IL‐1β and COX‐2 expression were significantly reduced by the combination therapies. In addition, a custom‐made nanostring panel demonstrated significant upregulation of genes participating in the formation of the extracellular matrix. Similarly, in the B16F10 melanoma model, mEHT and aspirin synergistically reduced the number of melanoma nodules in the lungs. In conclusion, mEHT combined with a selective COX‐2 inhibitor may offer a new therapeutic option in TNBC.

## Graphical abstract

The authors found that NSAIDs, such as aspirin and the selective COX‐2 inhibitor SC236, enhanced tumor growth inhibition and destruction induced by modulated electro‐hyperthermia by inhibiting the production of inflammatory cytokines.

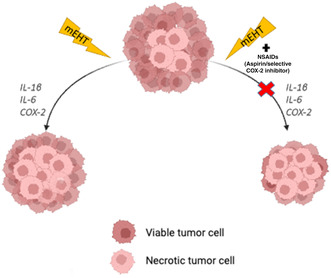

**Abbreviations**
APP: acute phase proteins
cC3: cleaved caspase‐3
COX: cyclooxygenase
DEG: differentially expressed genes
GO: Gene Ontology
H&E: hematoxylin and eosin
IHC: immunohistochemistry
IL1‐β: Interleukin 1 beta
IL‐6: interleukin 6
lAPR: local acute phase response
mEHT: modulated electro‐hyperthermia
NSAIDs: nonsteroid anti‐inflammatory drugs
TDR: tumor destruction ratio
TME: tumor microenvironment
TNBC: triple‐negative breast cancer

## 1 Introduction

Previously, we have demonstrated a strong antitumor effect of modulated electro‐hyperthermia (mEHT) treatment in the 4T1 triple‐negative breast cancer (TNBC) and in the B16F10 melanoma lung mouse models [1, 2]. The results of a multiplex analysis of the mEHT effects at both the gene and protein levels revealed a local acute phase response (lAPR): the synthesis of acute phase proteins (APP) (fibrinogen, haptoglobin, serpins, complement system proteins), and the optimization of the innate immune response in TNBC [3]. Here, we report that mEHT monotherapy in addition to the described reaction stimulates local inflammatory proteins such as interleukin‐1 Beta (IL‐1β), interleukin‐6 (IL‐6), and cyclooxygenase‐2 (COX‐2). The lAPR and the IL‐1β, IL‐6, and COX‐2 induction can be considered as part of a self‐defensive reaction of tumor cells due to mEHT‐induced stress, although the role of the inflammation in cancer is not fully understood [4, 5, 6, 7]. In the present *in vivo* study, mEHT was combined with nonsteroid anti‐inflammatory drugs (NSAIDs; aspirin (ASA) or SC236), which target multiple components of the acute phase reaction [8], and alone have already demonstrated promising outcomes in various tumors [9]. Here, we demonstrated that NSAID treatment synergistically increased the antitumor effect of mEHT in both 4T1 TNBC and B16F10 melanoma mouse models. Besides the inhibition of tumor growth, we have observed an increased tumor destruction ratio (TDR) in combined‐treated tumors, which was accompanied by an increase in cleaved caspase‐3 (cC3), suggesting that apoptosis plays an important role in the antitumor effect of mEHT and NSAIDs *in vivo*. To understand the molecular bases of synergism, we performed mRNA studies with RT‐PCR and nanostring. Molecular studies revealed a statistically significant reduction in the IL‐1β and COX‐2 expression by the combination therapy. Analysis of differentially expressed genes (DEG) using Gene Ontology (GO) through the Database for Annotation Visualization and Integrated Discovery (DAVID). Our findings suggest that the synergistic combination of mEHT and a selective COX‐2 inhibitor may regulate the extracellular matrix turnover and cell membrane protein expression.

Triple‐negative breast cancer and melanoma are aggressive, and recurring, with early metastasis compared with most other cancer types. TNBC accounts for 15% of all types of breast cancer. TNBC cells do not express the estrogen receptor, the progesterone receptor, or for the human epidermal growth factor receptor 2; consequently, there are no tumor‐specific (such as targeted or hormonal) treatment options, at present [10]. Contemporary treatments for melanoma include surgical resection, chemotherapy, and radiotherapy [11]. Thus, adjuvant treatments such as mEHT may aid TNBC and melanoma treatment [12, 13].

Modulated electro‐hyperthermia represents an adjuvant or supplementary treatment in different cancer models that are recently utilized in clinical medical practice [14]. mEHT contributes to tumor‐cell destruction selectively by accumulation of the radiation energy within the tumor without affecting healthy neighboring tissues [15]. Specific energy accumulation by the tumor cells is partially due to the altered (anaerobic) glucose metabolism of tumor cells (independent of the availability of oxygen called ‘Warburg effect’) [16].

Numerous epidemiological and experimental studies show that NSAIDs reduce the risk, incidence, and mortality in some cancers [17], including melanoma [18] and breast cancer [19]. Both nonselective (NSAIDs) and selective COX inhibitors (selCOXIBs) have been associated with lower cancer incidence; however, selCOXIBs have demonstrated greater significance than nonselective NSAIDs [20]. NSAIDs inhibit the production of prostaglandins, proinflammatory cytokines, and tumor growth factors [21, 22]. Furthermore, NSAIDs may affect COX‐independent inflammatory pathways such as NF‐κB [23], MAPK [24], mTOR [25], PDK‐1/Akt [26], and Wnt/β‐catenin [27], by the inhibition of activation of transcription factors. These pathways support cell proliferation and angiogenesis but suppress apoptosis, thus supporting tumor growth as well as participate in the regulation of the tumor microenvironment (TME). Therefore, COX inhibition influences tumor progression by decreasing migration [28], metastasis [29, 30], angiogenesis [31], increasing apoptosis, and sensitivity to other conventional anticancer therapies such as chemotherapy, immunotherapy, or radiotherapy [32, 33]. Besides the novel strategy of potential anticancer therapy, NSAIDs have a few adverse effects, such as gastrointestinal and cardiovascular complications as well as increased risk of renal or hepatic injury [34].

In this hypothesis, it is suggested that NSAIDs may regulate the TME and mEHT‐induced proinflammatory cytokines: IL‐1β, IL‐6, and COX‐2. As a result, NSAIDs may enhance the mEHT‐induced tumor cell death. Combining clinically available mEHT with NSAIDs is a new potential tool in oncologic therapy.

## 2 Materials and methods

### 2.1 Cell culture

B16F10 cells [B16‐F10 (RRID:CVCL_0159)] melanoma and 4T1 cells [4T1 (RRID:CVCL_0125)] TNBC mouse cell lines were purchased and cultured according to protocols described in the previous studies [1, 2]. Judy Lieberman (Lieberman Laboratory, Harvard University, Boston, MA, USA) provided the 4T1 cells, which were grown as adherent cultures in a medium (DMEM, 4.5 g·L^−1^ glucose without l‐glutamine and Phenol Red, Capricorn Scientific, Ebsdorfergrund, Germany) supplemented with 10% fetal bovine serum (FBS; South America Origin), EU approved, Euroclone S.p.A. Pero, Italy, l‐glutamine 200 mm (Capricorn Scientific, Ebsdorfergrund, Germany), and penicillin 100× (Capricorn Scientific, Ebsdorfergrund, Germany). ATCC (Manassas, VA, USA) provided the B16F10 mouse melanoma cell line (ATCC^®^CRL 6475TM). Cells were cultured in MEM supplemented with 1% (v/v) MEM‐vitamin solution, 5% (v/v) heat‐inactivated HyClone fetal bovine serum, 1 mm sodium pyruvate, 2 mm l‐glutamine, and 1% (v/v) nonessential amino acids (NEAAs) from Thermo Fisher Scientific (Waltham, MA, USA). All cell lines underwent regular mycoplasma screening, and all experiments were conducted using mycoplasma‐free cells. The cell lines underwent authentication in the past 3 years through multiple evaluations, comparing newly acquired data with well‐established databases and reference panels. The process ensures the ongoing verification and validation of cell line identities.

### 2.2 *In vivo* treatment of 4T1 TNBC

**Fig. 1:**
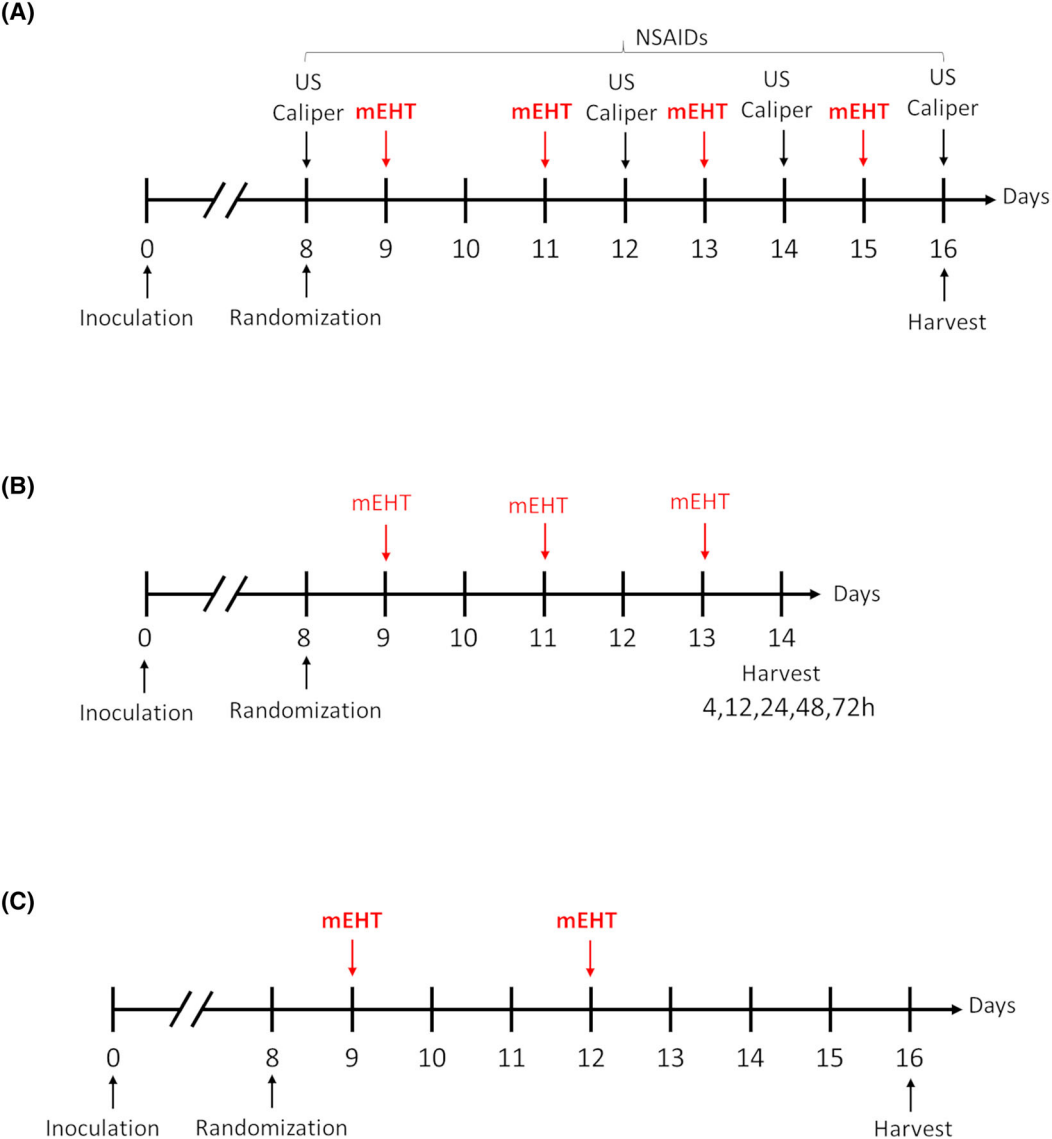
Experimental protocols of the 4T1 model. 4T1 TNBC cells were inoculated at Day zero. Mice were randomized at Day 8. mEHT treatments were performed every other day. NSAIDs were administered every day from randomization until the end of the experiment. Ultrasound and caliper tumor volume measurements were performed on every intermittent day. Tumors were harvested at the end of the study. (A) Combination therapy: 4× mEHT treatment in combination with aspirin or SC23. (B) Time‐kinetic experiment: 3× with mEHT treatment + tumor harvest at different time points. (C) Long‐term follow‐up: 2xmEHT treatment + tumor harvest at 96 h after the last treatment.

4T1 TNBC cells were cultured and prepared for inoculation. Using a Hamilton syringe (Hamilton Company, Reno, NV, USA), 1 × 10^6^ cells/50 L PBS (phosphate‐buffered saline with no magnesium and calcium, #17‐516F, Lonza A. G., Basel, Switzerland) were inoculated in the fourth mammary fat pad of 6‐ to 8‐week‐old female BALB/c mice. Female BALB/c mice were ordered from the National Institute of Oncology (Hungary) and were housed under minimal disease (MD) conditions at the Animal Facility of the Basic Medical Science Center of Semmelweis University with free access to standard mouse chow and tap water *ad libitum* and under a 12 h dark/light cycle. Animals were anesthetized with isoflurane (Baxter International Inc., Deerfield, IL, USA), 4–5% for induction, 1.5–2% to maintain anesthesia, and compressed air (0.4–0.6 L/min) for tumor‐cell injection. 4T1 tumor cells were subcutaneously injected into each mouse's inguinal mammary fat pad. On the eighth day after inoculation, tumor volume was measured using ultrasound and a digital caliper, as described earlier by Danics et al. [35]. Mice were randomized according to tumor volume into six different groups. On the same day, animals were given a daily dose of aspirin 100 mg·kg^−1^ (Sigma‐Aldrich Co., St. Louis, MO, USA) or SC236 6 mg·kg^−1^ (Axon Medchem BV, Groningen, The Netherlands.) via intraperitoneal injections [29]. Drugs were dissolved in 10% DMSO, 40% PEG300, 5% Tween‐80, and 45% saline. Treatment with COX inhibitors, administered every day during the entire experiment, and was combined with mEHT (Fig. 1A). Mice were treated four times every 48 h with a newly constructed labEHY‐200 (Oncotherm Ltd., Páty, Hungary) mEHT device, as detailed in our prior publications [1, 35]. Tumor volume was monitored by ultrasound and digital caliper every other day until the termination of the experiment. In two separate experiments, we investigated long‐term effects of mEHT treatment. In the time‐kinetic experiment (Fig. 1B), mice were treated with mEHT or Sham and tumors were harvested after 3 mEHT treatments at different time points. In the long‐term follow‐up experiment (Fig. 1C) tumors were harvested 96 h after two mEHT treatments. The mice were euthanized by cervical dislocation the next day after the last treatment. Tumors were excised, and they were cleaned for further processing. Half of the tumors were fixed in a 4% formaldehyde solution (Molar Chemicals Ltd., Halásztelek, Hungary), and the other half was cut into a few small pieces and stored at −80 °C for molecular studies. Interventions and animal housing were carried out in accordance with Hungarian Law Nos. XXVIII (1998 and 2002) and LXVII (2002), both of which deal with animal protection and welfare, as well as European Union guidelines. The National Scientific Ethical Committee on Animal Experimentation approved all animal treatments under the code PE/EA/50‐2/2019 on 1 November 2019.

### 2.3 *In vivo* treatment of B16F10 melanoma

**Fig. 2:**
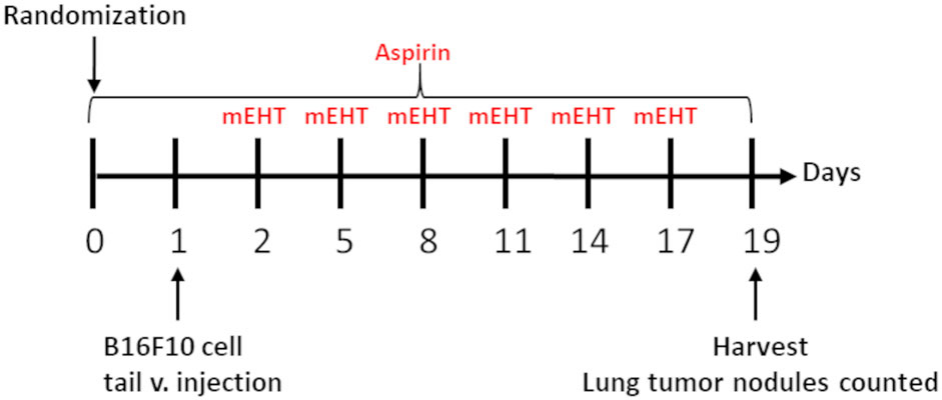
Experimental protocol of the B16F10 melanoma experiment. Randomization was performed at Day zero, B16F10 melanoma cells were inoculated through the tail vein at Day 1, mEHT treatments were performed at Days 2, 5, 8, 11, 14, and 17. Aspirin was administered every day from Day zero until the end of the experiment. Tumors were harvested at Day 19.

1 × 10^5^ B16F10 melanoma cells were injected into the tail vein of 7‐ to 9‐week‐old female C57BL/6 mice that have induced tumor nodules in the lungs. The next day after inoculation, mice were treated with mEHT alone or mEHT combined with aspirin 11.1 mmol·L^−1^ concentration [35] in drinking water [36]. Because of aspirin insolubility in water, first, aspirin was dissolved in DMSO 0.2% and then mixed with the drinking water using a magnetic mixer to mix it until a clear solution was achieved. Solution pH was adjusted to 7.4 physiological pH using a combination of NaOH and/or HCl and a pH meter. Animals have been treated six times using the LabEHY‐200 device set up to maintain 41–42 °C inside the mice's lungs [2]. The first mEHT treatment was performed 1 day after the tail vein inoculation of the B16F10 cells for 30 min, which was repeated six times in total on every third day (Fig. 2). Animals were terminated on Day 20, 48 h after the last mEHT treatment. The lung melanoma burden was assessed by counting the number of tumor nodules on the surface of the lungs [2].

### 2.4 RNA isolation and real‐time PCR

RNA was isolated using TRIzol reagent (Molecular Research Center Inc., Cincinnati, OH, USA) according to the manufacturer's instructions. A high‐capacity cDNA reverse transcription kit was used to reverse transcribe the isolated RNA (Applied Biosystems, Carlsbad, CA, USA). The amplified cDNA was used as template for the RT‐PCR. SYBR Green‐based RT‐PCR using Sso Advanced TM Universal SYBR^®^ Green Supermix and the CFX96 Touch Real‐Time PCR Detection System was used to detect messenger RNA in the samples (Bio‐Rad, Hercules, CA, USA). The 18S and GAPDH genes were used as housekeeping genes (Table 1).

**Table 1:** Primer pair designed for RT‐PCR.

Gene symbol	Gene name	Primer pair
18S	18S (Mus musculus)	Fwd: CTCAACACGGGAAACCTCAC Rev: CGCTCCACCAACTAAGAACG
GAPDH	Glyceraldehyde‐3‐phosphate dehydrogenase (Mus Musculus)	Fwd: CTCCCACTCTTCCACCTTCG Rev: GCCTCTCTTGCTCAGTGTCC
IL‐1β	Interleukin 1 beta (Mus Musculus)	Fwd: ACCTGTTCTTTGAGGCCGACA Rev: CCACAGCCACAATGAGTGAC
IL‐6	Interleukin 6 (Mus Musculus)	Fwd: GATGCTACCAAACTGGATATAATC Rev: GGTCCTTAGCCACTCCTTCTGTG
COX‐2	Ptgs2 (Mus Musculus)	Fwd: TCACGTGGAGTCCGCTTTAC Rev: AGGATGCAGTGCTGAGTTCC

### 2.5 Histopathology and immunohistochemistry

Tumor tissues were fixed in formalin and embedded in paraffin (FFPE). Using a polymer‐peroxidase system (Histols; Histopathology Ltd., Pécs, Hungary), serial sections (2.5 m) were sliced, dewaxed, and rehydrated for hematoxylin–eosin (HE) staining or immunohistochemistry (IHC) (Table 2) as described previously [1]. Viable tumor area per cross‐sectional tumor area was performed using QuantCenter image analysis software (3DHISTECH), and tumor TDR% was assessed as described previously [35].

**Table 2:** Antibodies and conditions used for IHC.

Antigen	Type	Reference no.	Dilution	Vendor
cCasp3	Rabbit, pAb	#9664	1 : 1600	Cell Signaling

### 2.6 Nanostring

Extracted RNA from *in vivo* experimental samples was used for RNA detection with NanoString technology. NanoString uses unique optical barcoded RNA that hybridizes to the target RNA in the sample to enable digital counting of individual RNA molecules without possible artifacts introduced by enzymatic steps. The gene expression panel (NanoString; Redwood, CA, USA) was custom‐made based on our previous publication in which mEHT‐regulated genes were detected by next‐generation sequencing (NGS) and verified by NanoString. The custom panel composed of 134 genes [1]. One hundred nanograms of RNA was used for hybridization. After hybridization, samples were transferred to the nCounter Prep Station for data collection on the nCounter Digital Analyzer. The 4.0 nsolver Analysis Software (NanoString, Redwood, CA, USA) was used for data analysis. Genes with log2 fold change values greater than 1.5 or less than −1.5 were considered the most regulated for further analysis. Values obtained from three replicates of two groups, mEHT or mEHT + SC236, were used to generate the volcano plot. DEGs were conducted utilizing the GO which was accessed through the DAVID [37]. GO analysis was used to identify genes that can be classified into different groups. In our study, we used the database for DAVID (https://david.ncifcrf.gov/) to perform functional annotation clustering the most regulated genes. The *P*‐value represents the probability of chance association between genes and a specific functional category. The *P*‐value was adjusted with the Benjamini–Hochberg (shortly: Benjamini) procedure to control for false discovery rate (FDR) by correcting for multiple comparisons.

### 2.7 Statistical data analysis

The graphpad prism software (v.6.01; GraphPad Software, Inc., La Jolla, CA, USA) was used for statistical analysis. Unpaired Mann–Whitney nonparametric tests were performed to compare the Sham and the mEHT‐treated groups. Follow‐up examinations were statistically evaluated with a one‐way ANOVA. Differences were considered statistically significant as *P < 0.05, **P < 0.01, ***P < 0.001, ****P < 0. 0001. Data are presented as mean ± SEM.

### 2.8 Ethics approval

The studies did not involve human participants, human data, or human tissue. Interventions and housing of the animals conformed to the Hungarian Laws No. XXVIII/1998 and LXVII/2002 about the protection and welfare of animals, and the directives of the European Union. All animal procedures were approved by the National Scientific Ethical Committee on Animal Experimentation under the No. PE/EA/50‐2/2019.

## 3 Results

### 3.1 mEHT‐induced IL‐1β, IL‐6, and COX‐2 mRNA was inhibited by NSAIDs

**Fig. 3:**
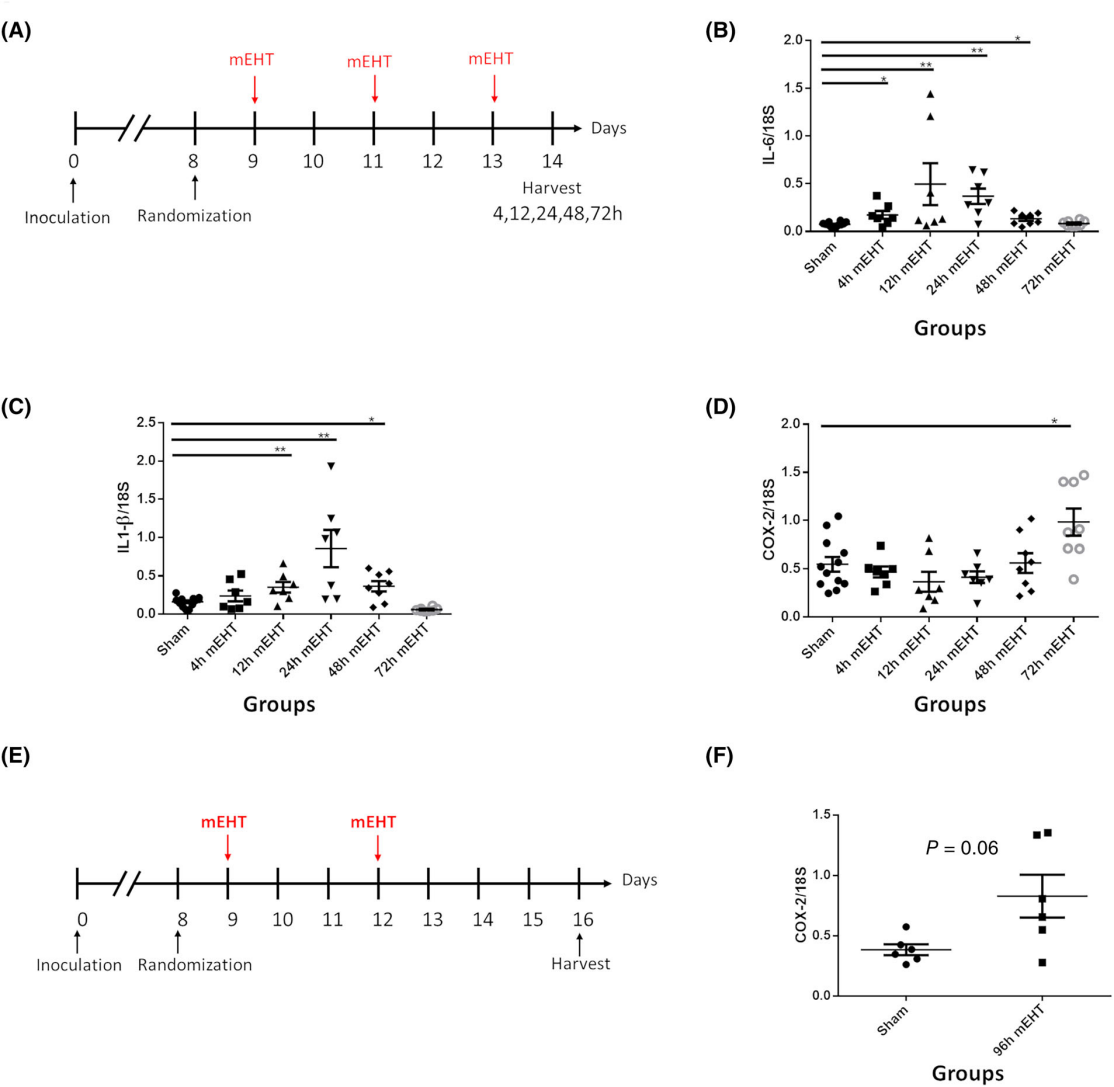
Time kinetics of proinflammatory cytokines' mRNA after mEHT treatment. (A) Experimental protocol of the time‐kinetic experiment. (B–D) Mice were treated three times with mEHT and terminated at 4, 12, 24, 48, and 72 h after last treatment. Inflammatory cytokines expression was measured: (B) IL‐6. (C) IL‐1β. (D) COX‐2. (E) Protocol of the long‐term follow‐up experiment (F) Mice treated twice with mEHT and terminated at 96 h and COX‐2 expression was measured. (F) COX‐2 mRNA 96 h after two mEHT treatments (*P* = 0.06). Expression normalized to 18S reference gene. Mean ± SEM, Unpaired Mann–Whitney test *P < 0.05; **P < 0.01. Number of animals/group: (B–D) Sham‐each time point:12, mEHT‐4 h:6; 12, 24 h:7; 48, 72 h:8; (F) 96 h: Sham:6; mEHT:6.

Modulated electro‐hyperthermia induced proinflammatory cytokines (IL‐6 and IL‐1β) in TNBC *in vivo*. In the time‐kinetic experiment, mice were terminated after the last mEHT treatment at 4 h, 12 h, 24 h, 48 h, and 72 h (Fig. 3A). IL‐6 (Fig. 3B) peaked at 12 h (*P* = 0.07), while IL‐1β (Fig. 3C) peaked at 24 h after the last mEHT treatment (*P* = 0.009). IL‐6 was 3.8‐ and IL‐1β three times higher in the mEHT‐treated mice vs Sham. COX‐2 mRNA was significantly elevated later, at 72 h post‐mEHT (Fig. 3C), which was preserved by 96 h after mEHT (Fig. 3D).

**Fig. 4:**
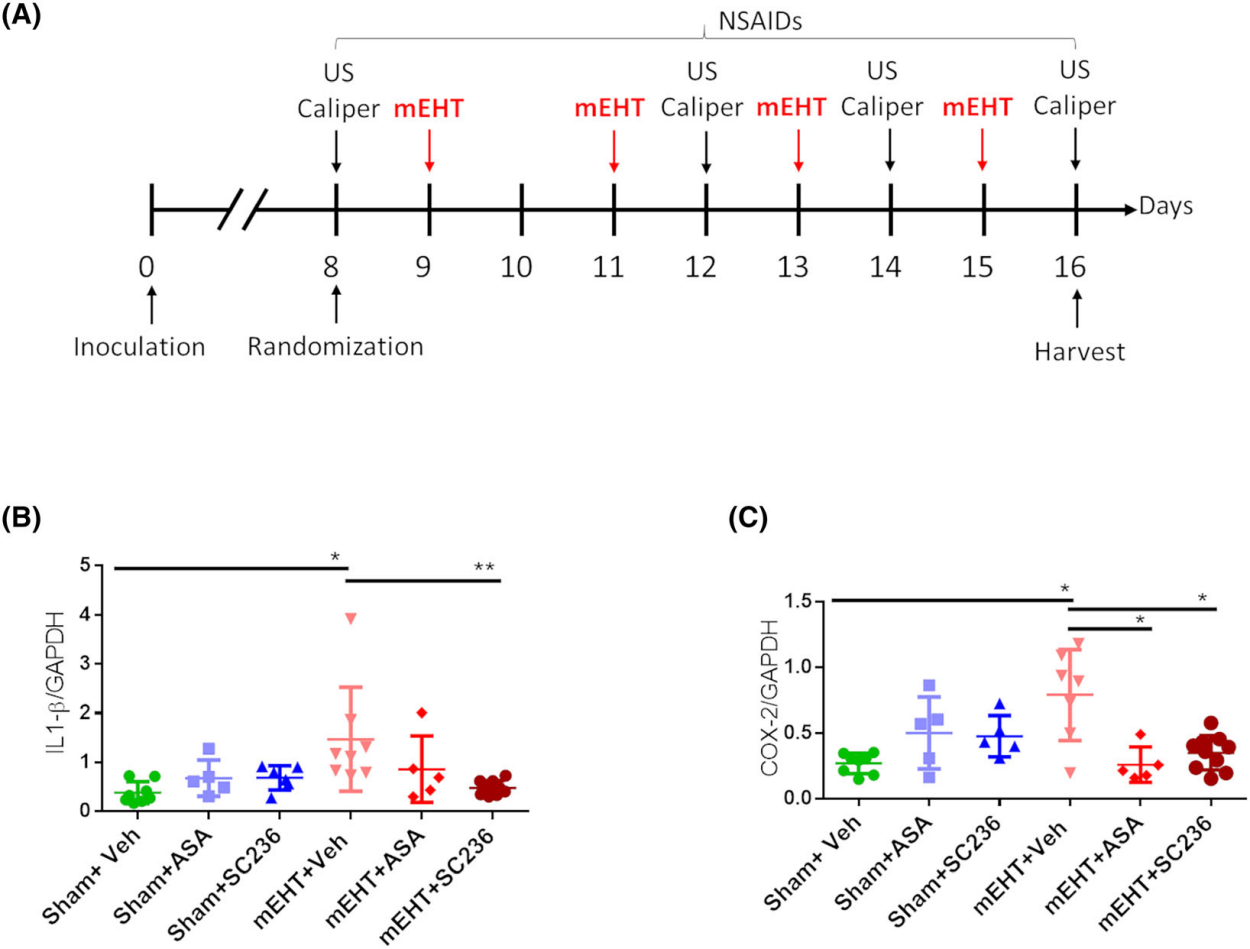
Proinflammatory cytokine mRNA after 4× mEHT and NSAID treatment. (A) Experimental protocol. (B) IL‐1β. (C) COX‐2. Expressions are normalized to GAPDH. One‐way ANOVA, Mean ± SEM, *P < 0.05; **P < 0.01. Number of animals/group: (B, C) Sham + Veh:6; Sham + ASA:5; Sham + SC236:5; mEHT + Veh:8; mEHT + ASA:5; mEHT + SC236:10.

In a separate experiment, mEHT and NSAID combination therapy was investigated (Fig. 4A). IL‐1β 3.8x (*P* = 0.002) and COX‐2 2.5x (*P* = 0.0002) mRNA were upregulated 24 h after 4 mEHT treatments. IL‐1β induction was almost completely reversed to the Sham level only by SC236 (*P* = 0.004) (Fig. 4B), whereas COX‐2 induction was reversed by both SC236 (*P* = 0.001) and aspirin (*P* = 0.001) (Fig. 4C).

### 3.2 mEHT‐inhibited tumor growth was accelerated by NSAID co‐treatment

**Fig. 5:**
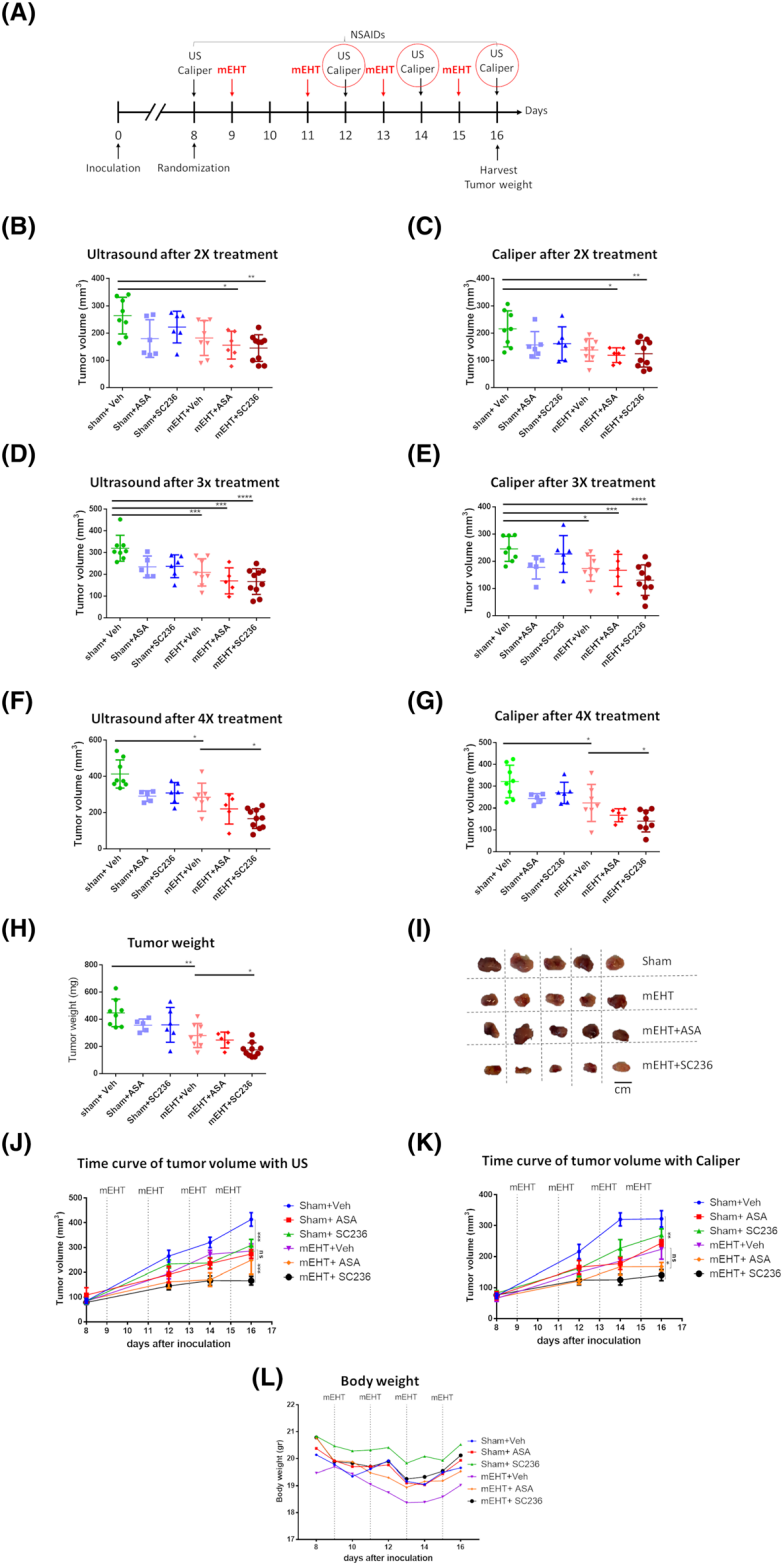
Tumor growth, tumor weight, and bodyweight. (A) Experimental protocol. Red circles: US + caliper data taken after the second, third, and fourth mEHT are given on Fig. 5B–G. Tumor volume after (B, C) 2, (D, E) 3, and (F, G) 4 mEHT treatments. (H) Tumor weight (milligrams) at the end of the experiment. (I) Pictures of all excised tumors (Scale bar, 1 cm). Time curves of tumor growth measured by (J) ultrasound and (K) caliper. (L) Body weight (grams). One‐way ANOVA, Mean ± SEM, *P < 0.05; **P < 0.01; ***P < 0.001; ****P < 0.0001. Number of animals/group: (B–H, J, K, L) Sham + Veh:8; Sham + ASA:5; Sham + SC236:5; mEHT + Veh:8; mEHT + ASA:5; mEHT + SC236:10.

During the mEHT and NSAID combination therapy experiment (Fig. 5A), tumor volumes were assessed by ultrasound and digital caliper. Tumor volumes were similar at randomization: 81.8 ± 17 mm^3^ (Fig. 5J,K). Tumor volumes progressed in Sham + vehicle‐treated mice from 264 ± 67 mm^3^ (after two treatments) to 320 ± 59 mm^3^ (after three treatments) and to 413 ± 77 mm^3^ (after four mEHT treatments) (Fig. 5B–G, J–K). Monotherapy mEHT (+vehicle) was able to significantly reduce tumor size only after 3 mEHT treatments (*P* = 0.06) (Fig. 5D,E). Average volume in the mEHT‐treated group was 258 ± 54 mm^3^ compared with 320 ± 59 mm^3^ in the Sham‐treated group. However, the combination therapy (mEHT + ASA/SC236) was able to significantly reduce tumor volume already after 2 mEHT treatments to 156 ± 51 mm^3^ (*P* = 0.02) and 145 ± 49 mm^3^ (*P* = 0.02) compared with the Sham‐treated group with an average volume of 264 ± 67 mm^3^ (Fig. 5B,C). After four treatments, only the COX‐2‐specific combination (mEHT + SC236) with 166 ± 54 mm^3^ average volume was significantly more effective than mEHT monotherapy—average volume 285 ± 77 mm^3^ (*P* = 0.02). The COX‐2‐specific combination (mEHT + SC236) was the most effective inhibitor of tumor growth (Fig. 5J,K). This observation was supported by significant reduction in the tumor weight at the end of the study (Fig. 5H,I). Average tumor weight in the mEHT‐treated group was 284 ± 88 mg, while in the mEHT + SC236, it was only 175 ± 51 mg (*P* = 0.04).

Most mice lost not more than 5–10% bodyweight by the third treatment; however, they recovered by the end of the study. The body weight loss was not significantly different between the groups. Sham‐treated mice (20.1 ± 1 g) lost an average 0.5 grams (19.6 ± 0.6 g) by the third treatment. The mEHT‐treated group (19.4 ± 1.5 g) lost an average 1.7 g (17.7 ± 1.6 g). Animals that received the combination treatment of mEHT and ASA (20.7 ± 0.7 g) lost only 1.2 g (19.5 ± 0.6 g), while those treated with mEHT + SC236 (20.7 ± 1 g) did not lose weight (Fig. 5L). Bodyweight by the end of the study did not differ significantly from the initial bodyweight in any of the groups.

### 3.3 mEHT‐induced tumor tissue destruction appeared mainly as cC3‐dependent apoptosis and was enhanced by NSAIDs

**Fig. 6:**
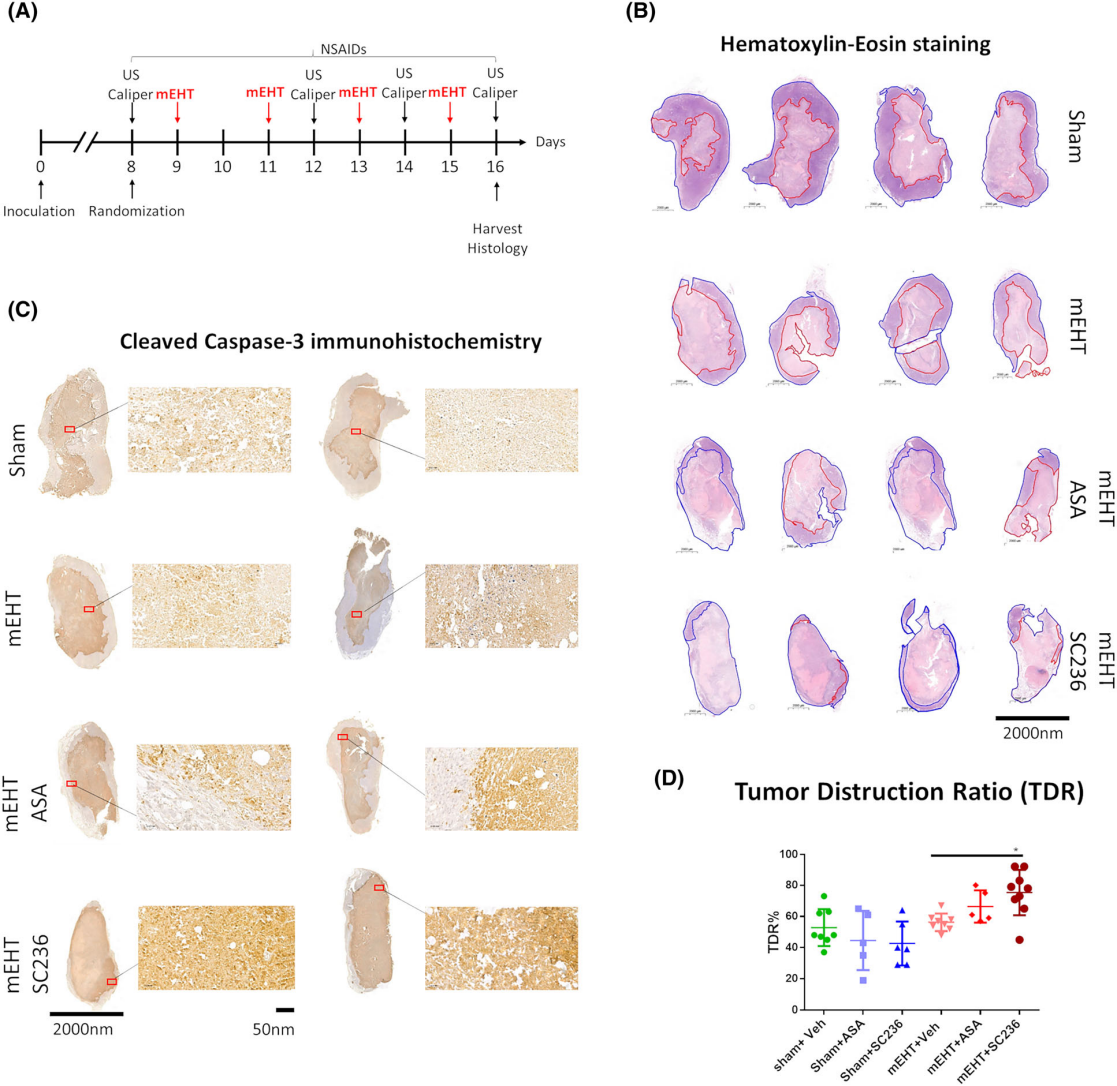
TDR% and cC3 staining in harvested tumors. (A) Experimental protocol. (B) Representative pictures of HE‐stained tumors–TDR% (red annotation) of whole tumors (blue annotation). (C) Representative pictures of cC3 stained tumors. (D) Quantification of TDR%. (Magnification: 40×) One‐way ANOVA, Mean ± SEM, *P < 0.05. Number of animals/group: (D) Sham + Veh:8; Sham + ASA:5; Sham + SC236:6; mEHT + Veh:8; mEHT + ASA:5; mEHT + SC236:9.

HE staining was performed after the mEHT and NSAID combination therapy experiment (Fig. 6A). Pale areas (dead cells) on the HE‐stained slides (Fig. 6B) corresponding to cC3 positive areas on the IHC slides (Fig. 6C) designated as destructed areas appeared on Sham‐treated tumors (TDR = 52 ± 11%). mEHT monotherapy (TDR = 56 ± 5%, *P* = 0.9) or in combination with aspirin (TDR = 66 ± 10%, *P* = 0.4) increased the TDR% to some extent; however, these did not reach statistical significance. Significant increase in the TDR was only achieved in the group treated with mEHT + SC236 (TDR = 75 ± 14%, *P* = 0.4). The TDR in the mEHT + SC236 group (TDR = 75 ± 14%) was significantly (*P* = 0.04) different also from the mEHT monotherapy (TDR = 56 ± 5%) group (Fig. 6D).

### 3.4 Multiplex (nanostring) analysis demonstrated that COX‐2 inhibition negatively correlated with tumor‐promoting factors associated with tumor‐cell membrane and extracellular matrix

**Fig. 7:**
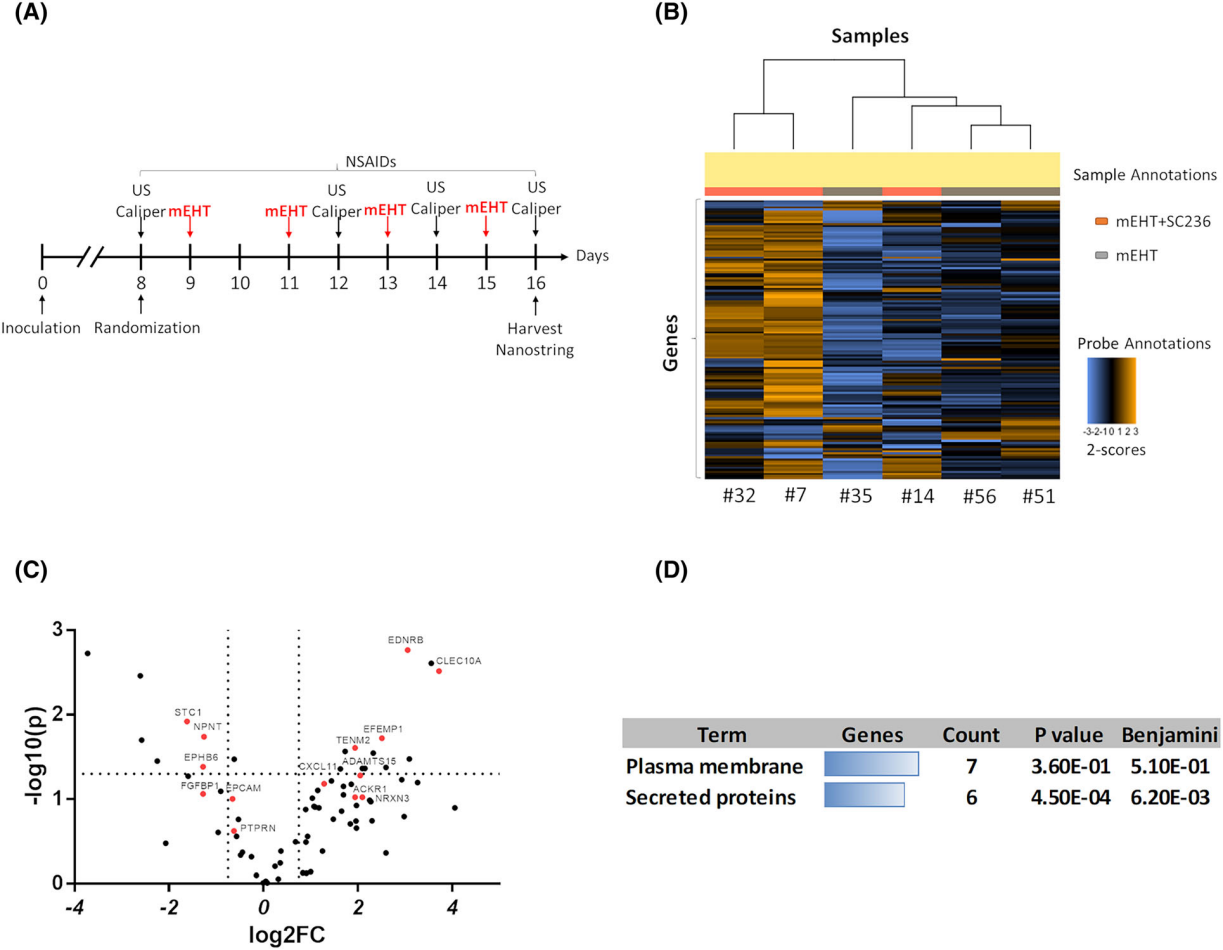
Heat map and volcano plot of DEGs after modulated electro‐hyperthermia (mEHT) vs mEHT + SC236 treatment from nanostring data. (A) Experimental protocol. (B) Heat map of 74 DEGs (nanostring nSolver® advanced analysis). Brown indicates upregulation, and blue indicates downregulation. (C) −10log(p) plotted against fold changes (log_2_FC). Vertical dotted lines: log_2_FC = 0.75, horizontal dotted line: −10log(p) = 1.30103. *n* = 3/group. Genes marked with red dots are identified in Table 3. (D) Regulated genes were clustered into two groups by DAVID. The Benjamini‐adjusted *P*‐value indicates enrichment of genes in a particular functional category.

Samples from the mEHT + NSAIDs experiment (Fig. 7A) passed the nanostring quality control (QC) and the nanostring run was successful. Seventy‐four identified DEGs are displayed on the heat map (Fig. 7B). Most regulated genes are presented on the volcano plot as well (Fig. 7C). Genes were identified (Table 3) and clustered in two groups using DAVID. Seven genes were identified as membrane proteins and six genes as secreted proteins. The *P*‐value established with the Benjamini procedure is also given on (Fig. 7D).

**Table 3:** Absolute mRNA count of secreted and membrane proteins from the nanostring data. Individual data of the mEHT and mEHT + SC236 group members and group averages. Bold indicates downregulation, and nonbold indicates upregulation. Linear mRNA data were received through the nSolver advanced analysis method. Cell line 4T1. The underlined values are the average value of the 3 samples (as the label of the column). Avg., average shows.

Group	RNA count	mEHT				mEHT + SC236			
	Genes	#35	#51	#56	Avg.	#7	#14	#32	Avg.
Secreted proteins	*Adamts15*	*28*	*98*	*74*	*65*	*294*	*286*	*113*	*231*
	*Cxcl11*	*77*	*89*	*148*	*105*	*370*	*213*	*396*	*326*
	*Efemp1*	*51*	*118*	*150*	*106*	*940*	*562*	*325*	*609*
	**Fgfbp1**	**355**	**224**	**155**	**245**	**56**	**174**	**68**	**99**
	**Npnt**	**1870**	**1714**	**817**	**1467**	**499**	**610**	**762**	**323**
	**Stc1**	**544**	**464**	**232**	**413**	**138**	**102**	**190**	**143**
Membrane proteins	*Ackr1*	*24*	*46*	*22*	*31*	*143*	*136*	*42*	*107*
	*Tenm2*	*3*	*19*	*19*	*14*	*47*	*87*	*35*	*56*
	*Clec10a*	*48*	*98*	*80*	*75*	*1239*	*1173*	*418*	*943*
	*Ednrb*	*48*	*62*	*51*	*54*	*614*	*352*	*231*	*399*
	*Nrxn3*	*4*	*10*	*11*	*9*	*27*	*40*	*22*	*29*
	**Ephb6**	**75**	**51**	**52**	**59**	**22**	**44**	**24**	**30**
	**Epcam**	**3263**	**5624**	**4580**	**4489**	**1832**	**3646**	**3090**	**2856**

Upregulated proteins in mEHT + SC236 vs mEHT monotherapy were as follows:

- A disintegrin‐like and metalloproteinase—with thrombospondin type 1 motif 15 (ADAMTS15); C‐X‐C motif chemokine Ligand 11 (CXCL11); EGF‐containing fibulin‐like extracellular matrix protein (EFEMP1), which were identified by DAVID as secreted proteins.
- Atypical chemokine receptor 1 (ACKR1); teneurin transmembrane protein 2 (TENM2); C‐type lectin domain‐containing (CLEC10A); endothelin B receptor (EDNRB); neurexin protease −3 (NRXN3), which were identified by DAVID as membrane proteins.

Downregulated proteins were as follows:

- Fibroblast growth factor‐binding protein (FGFBP); nephronectin (NPNT); Stanniocalcin‐1 (STC1), which were identified by DAVID as secreted proteins.
- Epithelial cell adhesion molecule (EPCAM) and ephrin type‐B receptor 6 (EPHB6), which were identified by DAVID as membrane proteins.

### 3.5 Aspirin diminished lung nodules in the B16F10 melanoma model

**Fig. 8:**
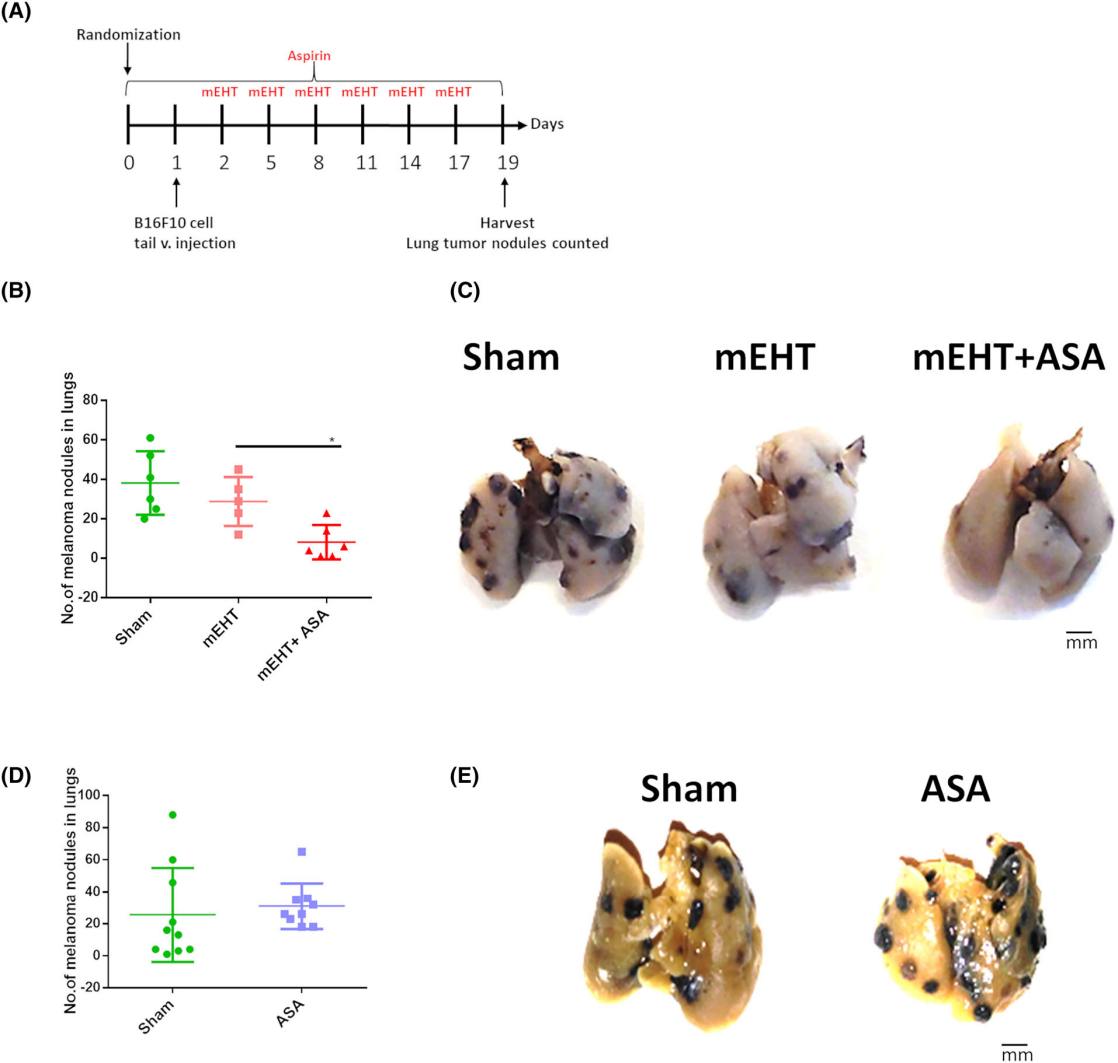
Effect of mEHT in combination with aspirin on tumor burden in B16F10 mouse model. (A) Experimental protocol. (B) Quantification and (C) representative images of melanoma lung nodules in Sham, mEHT, and Aspirin + mEHT groups and in Sham and aspirin groups (D, E). (Scale bar, 1 mm). Mean ± SEM, Unpaired Mann–Whitney test *P < 0.05. Number of animals/group: (B) Sham:6; mEHT:5; mEHT + ASA:6; (D) Sham:10; ASA:9.

In the melanoma tail vein injection model (Fig. 8A), pulmonary melanoma nodules were counted. In untreated (Sham) lungs, 38.1 ± 16 nodules were counted. mEHT treatment alone reduced the number of nodules (28.8 ± 12) although the difference was not statistically significant (*P* = 0.4). However, mEHT combined with aspirin, significantly (*P* = 0.02) decreased the number of nodules compared with mEHT alone (8.1 ± 8) (Fig. 8B,C). On the contrary, aspirin alone (31 ± 29) had no significant effect compared with Sham (26 ± 14) (Fig. 8D,E).

## 4 Discussion

In the present study, we demonstrated that mEHT monotherapy induced inflammatory cytokines such as IL‐6 and IL‐1β. Inflammatory cytokines are a sign of more aggressive cancer phenotype [38, 39]. By creating an inflammatory microenvironment within the tumor, IL‐6 and IL‐1β may promote tumor‐cell proliferation by activation of transcription factors and angiogenesis by the induction of key factors such as vascular endothelial growth factor (VEGF). Mutant cells generate cytokines and attract inflammatory cells [40]. COX‐2 has a strong relationship with IL‐6 and IL‐1β [41]. Our data revealed a COX‐2 induction 72‐ and 96‐h after mEHT treatment, which may be explained by the earlier upregulation of IL‐6 and IL‐1β at 12 h and 24 h. Inflammatory COX‐2 has been demonstrated to be induced by IL‐1β [42] or IL‐6 [43]. IL‐6 has been proposed as an important tumor‐promoting cytokine [44]. Similarly, to our results a recent paper demonstrated relatively late COX‐2 induction at 24 h by chemotherapy in 4T1 cells [45]. In that study, COX‐2 inhibition was essential for effective combination therapy (chemo + immune therapy), similar to our study. Furthermore, previous studies demonstrated that other anticancer therapies, such as chemo‐ and photodynamic therapies have also induced significant IL‐1β and COX‐2 expression activating the tumor‐favorable microenvironment. These studies have also utilized COX‐2 inhibitors to overcome this problem. A combination of photodynamic therapy with selective COX‐2 inhibitors effectively inhibited inflammatory cytokine and VEGF synthesis and enhanced therapy‐induced cytotoxicity and apoptosis [46]. Similarly, co‐administration of COX‐2 inhibitors with chemotherapy or ablation of COX‐2 with CRISPR effectively enhanced the treatment efficacy by reducing production of inflammatory proteins [45].

We combined mEHT with aspirin or the selCOXIB SC‐236 in TNBC 4T1 and B16F10 melanoma experimental mouse models. The 4T1 TNBC growth inhibitory effect of mEHT monotherapy was quite similar to our previously published studies, where mEHT alone effectively inhibited 4T1 TNBC growth after the third treatment [1, 35]. However, here the combination of mEHT with NSAIDs demonstrated significant tumor shrinkage earlier: already after the second treatment, as revealed by both ultrasound and a digital caliper measurements of tumor volume. The clinical relevance of achieving tumor reduction at an earlier stage of treatment is enormous as earlier inhibition of exponential tumor growth offers better prognosis. Shrinking the tumor early may render it more susceptible to subsequent treatments such as surgery, radiation, or chemotherapy. Thus, early tumor size reduction could potentially make these treatments more effective, leading to better outcomes [47]. Early cancer treatment is also emphasized by the recent NHS cancer guideline [48]. After the fourth treatment only the SC236 combination group reached statistical significance when compared to mEHT. A previous study demonstrated that in CT26 colon cancer, mEHT efficacy was significantly enhanced by curcumin, with known anti‐inflammatory and antioxidant properties [49].

Neither mEHT nor the NSAIDs nor any combination appeared to be toxic based on the body weight data as animals did not lose more than 5–10% of their body weight [47]. Assessing treatment toxicity in preclinical models is crucial to evaluate the safety for clinical translation [50]. Based on the body surface area dose conversion method [51], 100 mg·kg^−1^ ASA is equivalent to a moderate human ASA dose of 300–500 mg·day^−1^ [28]. Long‐term ASA treatment can result in renal, cardiovascular, and gastric toxicity as well as bleeding and hypersensitivity [52]. SC236 used in the present study is analogous to celecoxib. Celecoxib is associated with a decreased risk of gastrointestinal bleeding, although it still has a higher incidence of cardiovascular events in comparison with traditional NSAIDs [53]. Although the applied NSAID dose + mEHT combination did not appear to have any toxicity, the toxicity findings of the present mouse study must be reevaluated in large animal models and early‐phase clinical studies before reaching conclusions relevant for the clinical setting.

Besides the tumor size reduction in the NSAID combined groups, the TDR of the remining small tumors was also larger. However, in mEHT‐monotherapy‐treated tumors, only tumor size was smaller than in the Sham‐treated tumors; the TDR did not differ significantly, similarly to our previous studies [1]. The lack of significant increase in TDR is largely due to the severe spontaneous destruction of Sham tumors growing to a size where spontaneous necrosis is evident [54].

In addition, the tumor damage response was accompanied by a significant increase in cC3, suggesting that apoptosis played an important role in the antitumor effect of mEHT and NSAIDs [55]. As cancer cells often evade apoptosis, inducing apoptosis in cancer cells is a critical goal of cancer treatment. The intrinsic resistance of cancer cells to apoptosis is the basis of resistance to chemotherapy [56]. Thus, induction or reactivation of apoptosis in cancer sensitizes cancer cells to therapy [57]. Monotherapy with both mEHT or COX‐2 inhibitors induced significant cC3 staining in TNBC in previous [33, 35] as well as in the present study. COX‐2‐and mEHT‐induced apoptosis may have contributed to the synergistic effects of mEHT + NSAIDs [17, 58]. According to our RT‐PCR data, NSAIDs attenuated mEHT‐induced IL‐1β and COX‐2 expression. COX‐2 and IL‐1β are regulated together [8]. Besides inflammatory proteins, NSAIDs may affect COX‐independent inflammatory pathways, by the inhibition of transcription factor activation of pathways, which support cell proliferation and angiogenesis but suppress apoptosis [23, 24, 25, 26]. Therefore, COX inhibition induced apoptosis sensitizes cancer to other conventional anticancer therapies such as chemotherapy, immunotherapy, radiotherapy [32, 33] or mEHT.

The results of the nanostring multiplex data analysis demonstrated that the combination of mEHT with a selective COX‐2 inhibitor regulated secreted and cell membrane proteins. Some of the secreted proteins are extracellular matrix (ECM) proteins (EFEMP1, NPNT, and STC), ECM regulators (ADAMTS15, CXCL11, and FGFBP1) and thus are an essential part of the TME. The identified membrane proteins serve as receptors (EPHB6, ACKR1, and EDNRB) or adhesion molecules (EPCAM) and may enhance antitumor immunity (TENM2 and CLEC‐10A); thus, they may contribute to enhanced mEHT‐induced cancer destruction through regulating the TME related immune response.

ADAMTS15 was upregulated in mEHT+ selCoxIb (SC236) vs mEHT monotherapy. Its biological significance is its involvement in extracellular matrix remodeling [59, 60]. ADAMTS15 participates in tissue organization and vascular homeostasis. ADAMTS15 is a secreted protease modifying the extracellular matrix components like proteoglycans and collagen [61]. ADAMTS15's role in tumor pathophysiology is inhibition of angiogenesis and cell migration [62]. To the best of our knowledge, the regulation of ADAMTS15 by COX inhibition has not been described before. ADAMTS15 may be acting as a tumor suppressor in breast cancer by modulating cell‐environment interactions [63, 64]. ADAMTS15 expression is a favorable prognostic factor in breast cancer. Higher expression has been associated with better clinical outcomes, including longer overall survival and disease‐free survival [62]. As ADAMTS15 high levels are signs of good prognosis in breast cancer [65]. Thus, the observed upregulation could have contributed to the observed synergistic effects.

Similarly, secreted proteins, such as EFEMP1 and CXCL11, which are known targets of selCOXIBs [8], were also upregulated in the combination group. EFEMP1 is an extracellular matrix protein, associated with elastic fiber formation and cell adhesion [66] and is considered as a tumor‐suppressor gene [67, 68]. Its expression is diminished in breast cancer [69]. In tumor biology, antiangiogenic properties (reduction in angiogenic sprouting) of EFEMP1 have been described [70]. Although contradictory data suggesting EFEMP1 to be a bad prognostic indicator have been also published [71], our data support the beneficial role of EFEMP1 in breast cancer. CXCL11 is involved in the immune response including T‐cell and TME regulation. CXCL11 can enhance antitumor immune cell migration and infiltration in the breast cancer tissue [72]. In our study, upregulation of EFEMP1 and CXCL11 proteins could have contributed to the synergistic effect between mEHT and the selCOXib therapy.

Additionally, downregulated secreted glycoproteins were FGFBP1, STC1, and NPNT. FGFBP1's physiologic role is regulating fibroblasts and some cellular processes, such as differentiation and growth. In cancer biology, FGFBP1 may stimulate angiogenesis, PD‐L1 expression, and immune inhibition [73, 74]. STC1 is a secreted glycoprotein. Its biological significance is the regulation of phosphate and calcium homeostasis [75]. In cancer, STC1 enhances metastasis via the PI‐3K/Akt/NF‐kB signaling pathways [76]. Similar to our results, a study demonstrated that NSAIDs (Ibuprofen) attenuated IL‐1β‐induced STC1 expression in chondrocytes [77]. NPNT is a secreted glycoprotein into the TNBC extracellular matrix and plays a role in adhesion and migration. In cancer, NPNT may promote metastasis via its integrin‐binding site, which is important for adhesion and transmigration through the endothelial cells [78]. Thus, downregulation of NPNT by NSAIDs may have contributed to the reduced tumor formation in our lung melanoma model. Regulation of NPNT and FGFBP1 by COX inhibition has not been described before. As described, FGFBP1, STC1, and NPNT proteins have tumor‐promoting effect thus, downregulation of these proteins could have contributed to the synergistic effect in our study.

Membrane glycoproteins such as ACKR1, EDNRB, TENM2, and CLEC‐10A were highly expressed in the selCoxIb‐treated group. These proteins are considered as predictor biomarkers of good prognosis of various cancers. They may negatively regulate the tumor‐favorable microenvironment and support the antitumor immunity in breast cancer, although their role in tumor pathophysiology is not yet completely understood [79, 80, 81, 82]. The regulation of these proteins by COX inhibition has not been described before. In our study, the upregulation of these proteins by selCoxIb probably contributed to the enhanced effects of mEHT in treated tumors.

Neurexin 3 (NRXN3) encodes a protein involved in synaptic signaling and neural development in the nervous system [83]. Overlaps between genes involved in neural development and in cancer suggest links between nervous system development and tumorigenesis [84, 85]. Emerging research suggests a role of NRXN3 in cancer progression, including breast cancer [86], through its involvement in cellular adhesion, migration, and invasion.

In addition, membrane proteins, EPCAM, and EPHB6 gene expression were downregulated by selCoxIb treatment. Their biological role has been associated with cell adhesion and signaling. Thus, they may stimulate 4T1 cell growth; therefore, EPCAM and EPHB6 are associated with poor survival [87]. Silencing EPCAM significantly decreased the capacity of TNBC cell proliferation [88]. EPCAM expression is related to COX‐2 expression, TME regulation, and angiogenesis [89]. Downregulation of EPCAM and EPHB6 glycoproteins could contribute to the positive effects of the addition of selCoxIb to mEHT in 4T1 TNBC.

The listed regulated genes are mostly considered predictor biomarkers of prognosis in various cancers. The clinical implications of these genes are often context‐dependent, and continuous research is essential to comprehend their roles in diseases and how they can be utilized for clinical purposes such as diagnosis, prognosis, and targeted therapies.

Furthermore, we investigated the combination therapy of mEHT with aspirin in the B16F10 melanoma model. Tumor nodules in the lungs were targeted with a new application method developed by Thomas [2]. The pulmonary nodules were counted to evaluate the effect of the combination therapy. Our findings suggest that mEHT efficacy was enhanced by aspirin. Aspirin alone did not reduce the nodule count in the lungs. A Previous studies have demonstrated beneficial effects of aspirin in the B16F10 tumor model, by the induction of cC3‐mediated apoptosis [30, 36]. The role of cC3‐mediated apoptosis is supported by our study and cC3‐mediated apoptosis may play a role in the synergistic tumor growth inhibition observed by us [34, 35].

Limitations of the study include the lack of human data. Although mEHT has been extensively applied in the clinical practice in several countries, its combination with selective COX2 inhibitors or nonselective nonsteroidal anti‐inflammatory drugs has not been tested before. Further investigation of the role of the proteins identified by nanostring as potential mediators of the synergy between mEHT and selective COX2 inhibition are potential avenues for future research.

## 5 Conclusion

We discovered that mEHT treatment stimulated the expression of proinflammatory cytokines IL1‐β, IL‐6, and COX‐2 in TNBC. Our animal studies investigating the mEHT and NSAID combination therapy demonstrated a synergistic inhibition of tumor growth in both 4T1 TNBC and B16F10 melanoma cancer models. RT‐PCR and nanostring analysis suggested that therapeutic combination attenuated the mEHT‐induced IL1‐β and COX‐2 stimulation. In addition, selCoxibs may modulate the extracellular matrix, and cell membrane function in the TME leading to inhibition of cancer cell proliferation. Thus, this new combination treatment protocol could be implicated in the clinical setting as a therapeutic option due to its effectiveness and availability. Using the combination of mEHT with COX inhibitors may offer a new therapeutic possibility for various cancer types.

## Conflict of interest

The authors declare no conflict of interest.

## Author contributions

NG conceived and designed the study under the supervision of PH, NG, PHLV, CAS, KA, SMZB, JMT, EM, DB, and SMZK performed the animal experiments and processed the harvested animals for further analysis. ZB contributed to the financing, initiation, and theoretical supervision of the project. All authors reviewed and approved the manuscript.

### Peer review

The peer review history for this article is available at https://www.webofscience.com/api/gateway/wos/peer‐review/10.1002/1878‐0261.13585.

## Data Availability

All data associated with this study are presented in the paper. The data that support the findings of this study are available from the corresponding author upon reasonable request.

## References

[1] Schvarcz CA , Danics L , Krenács T , Viana P , Béres R , Vancsik T , et al. Modulated electro‐hyperthermia induces a prominent local stress response and growth inhibition in mouse breast cancer isografts. Cancers. 2021;13(7):1744. 10.3390/cancers13071744 33917524 PMC8038813

[2] Thomas MJ , Major E , Benedek A , Horváth I , Máthé D , Bergmann R , et al. Suppression of metastatic melanoma growth in lung by modulated electro‐hyperthermia monitored by a minimally invasive heat stress testing approach in mice. Cancers. 2020;12(12):1–24. 10.3390/cancers12123872 PMC7767533 33371498

[3] Hamar P . A new role of acute phase proteins: local production is an ancient, general stress‐response system of mammalian cells. Int J Mol Sci. 2022;23(6):2972. 10.3390/ijms23062972 35328392 PMC8954921

[4] Sinha P , Clements VK , Fulton AM , Ostrand‐Rosenberg S . Prostaglandin E2 promotes tumor progression by inducing myeloid‐derived suppressor cells. Cancer Res. 2007;67(9):4507–4513. 10.1158/0008-5472.CAN-06-4174 17483367

[5] Bunt SK , Sinha P , Clements VK , Leips J , Ostrand‐Rosenberg S . Inflammation induces myeloid‐derived suppressor cells that facilitate tumor progression. J Immunol. 2006;176(1):284–290. 10.4049/jimmunol.176.1.284 16365420

[6] An J , Feng L , Ren J , Li Y , Li G , Liu C , et al. Chronic stress promotes breast carcinoma metastasis by accumulating myeloid‐derived suppressor cells through activating β‐adrenergic signaling. Onco Targets Ther. 2021;10(1):2004659. 10.1080/2162402X.2021.2004659 PMC8632282 34858728

[7] Hong DS , Angelo LS , Kurzrock R . Interleukin‐6 and its receptor in cancer: implications for translational therapeutics. Cancer. 2007;110(9):1911–1928. 10.1002/cncr.22999 17849470

[8] Zelenay S , van der Veen AG , Böttcher JP , Snelgrove KJ , Rogers N , Acton SE , et al. Cyclooxygenase‐dependent tumor growth through evasion of immunity. Cell. 2015;162(6):1257–1270. 10.1016/j.cell.2015.08.015 26343581 PMC4597191

[9] Zhang Q , Zhu B , Li Y . Resolution of cancer‐promoting inflammation: a new approach for anticancer therapy. Front Immunol. 2017;8:71. 10.3389/fimmu.2017.00071 28210259 PMC5288347

[10] Kau P , Nagaraja GM , Zheng H , Gizachew D , Galukande M , Krishnan S , et al. A mouse model for triple‐negative breast cancer tumor‐initiating cells (TNBC‐TICs) exhibits similar aggressive phenotype to the human disease. BMC Cancer. 2012;12:120. 10.1186/1471-2407-12-120 22452810 PMC3340297

[11] Chattopadhyay C , Kim DW , Gombos DS , Oba J , Qin Y , Williams MD , et al. Uveal melanoma: from diagnosis to treatment and the science in between. Cancer. 2016;122(15):2299–2312. 10.1002/cncr.29727 26991400 PMC5567680

[12] Sood S , Jayachandiran R , Pandey S . Current advancements and novel strategies in the treatment of metastatic melanoma. Integr Cancer Ther. 2021;20:1534735421990078. 10.1177/1534735421990078 33719631 PMC8743966

[13] Yin L , Duan JJ , Bian XW , Yu SC . Triple‐negative breast cancer molecular subtyping and treatment progress. Breast Cancer Res. 2020;22(1):61. 10.1186/s13058-020-01296-5 32517735 PMC7285581

[14] Szasz AM , Minnaar CA , Szentmártoni G , Szigeti GP , Dank M . Review of the clinical evidences of modulated electro‐hyperthermia (mEHT) method: an update for the practicing oncologist. Front Oncol. 2019;9:1012. 10.3389/fonc.2019.01012 31737558 PMC6837995

[15] Krenacs T , Meggyeshazi N , Forika G , Kiss E , Hamar P , Szekely T , et al. Modulated electro‐hyperthermia‐induced tumor damage mechanisms revealed in cancer models. Int J Mol Sci. 2020;21(17):1–25. 10.3390/ijms21176270 PMC7504298 32872532

[16] Szigeti GYP , Szasz AM , Szasz O . Oncothermia is a kind of oncological hyperthermia–a review. Oncothermia J. 2020:8–48. [cited 2023 Feb 23]. Available from: https://www.cambridgescholars.com/product/978-1-5275-4817-6

[17] Kolawole OR , Kashfi K . NSAIDs and cancer resolution: new paradigms beyond cyclooxygenase. Int J Mol Sci. 2022;23(3):1432. 10.3390/ijms23031432 35163356 PMC8836048

[18] Gamba CA , Swetter SM , Stefanick ML , Kubo J , Desai M , Spaunhurst KM , et al. Aspirin is associated with lower melanoma risk among postmenopausal Caucasian women: the Women's Health Initiative. Cancer. 2013;119(8):1562–1569. 10.1002/cncr.27817 23483536 PMC3880825

[19] Bardia A , Keenan TE , Ebbert JO , Lazovich DA , Wang AH , Vierkant RA , et al. Personalizing aspirin use for targeted breast cancer chemoprevention in postmenopausal women. Mayo Clin Proc. 2016;91(1):71–80. 10.1016/j.mayocp.2015.10.018 26678006 PMC4807132

[20] Ashok V , Dash C , Rohan TE , Sprafka JM , Terry PD . Selective cyclooxygenase‐2 (COX‐2) inhibitors and breast cancer risk. Breast. 2011;20(1):66–70. 10.1016/j.breast.2010.07.004 20724158

[21] Hsieh CC , Huang YS . Aspirin breaks the crosstalk between 3T3‐L1 adipocytes and 4T1 breast cancer cells by regulating cytokine production. PLoS One. 2016;11(1):e0147161. 10.1371/journal.pone.0147161 26794215 PMC4721678

[22] Gilligan MM , Gartung A , Sulciner ML , Norris PC , Sukhatme VP , Bielenberg DR , et al. Aspirin‐triggered proresolving mediators stimulate resolution in cancer. Proc Natl Acad Sci U S A. 2019;116(13):6292–6297. 10.1073/pnas.1804000116 30862734 PMC6442621

[23] Park MH , Hong JT . Roles of NF‐κB in cancer and inflammatory diseases and their therapeutic approaches. Cell. 2016;5(2):15. 10.3390/cells5020015 PMC4931664 27043634

[24] Setia S , Nehru B , Sanyal SN . Upregulation of MAPK/Erk and PI3K/Akt pathways in ulcerative colitis‐associated colon cancer. Biomed Pharmacother. 2014;68(8):1023–1029. 10.1016/j.biopha.2014.09.006 25443414

[25] Zhang P , He D , Song E , Jiang M , Song Y . Celecoxib enhances the sensitivity of nonsmall‐ cell lung cancer cells to radiationinduced apoptosis through downregulation of the Akt/mTOR signaling pathway and COX‐2 expression. PLoS One. 2019;14(10). 10.1371/journal.pone.0223760 PMC6793859 31613929

[26] Benelli R , Barboro P , Costa D , Astigiano S , Barbieri O , Capaia M , et al. Multifocal signal modulation therapy by celecoxib: a strategy for managing castration‐resistant prostate cancer. Int J Mol Sci. 2019;20(23):6091. 10.3390/ijms20236091 31816863 PMC6929142

[27] Huang C , Chen Y , Liu H , Yang J , Song X , Zhao J , et al. Celecoxib targets breast cancer stem cells by inhibiting the synthesis of prostaglandin E2 and down‐regulating the Wnt pathway activity. Oncotarget. 2017;8(70):115254–115269. 29383157 10.18632/oncotarget.23250 PMC5777769

[28] Maity G , De A , Das A , Banerjee S , Sarkar S , Banerjee SK . Aspirin blocks growth of breast tumor cells and tumor‐initiating cells and induces reprogramming factors of mesenchymal to epithelial transition. Lab Invest. 2015;95(7):702–717. 10.1038/labinvest.2015.49 25867761

[29] Roche‐Nagle G , Connolly EM , Eng M , Bouchier‐Hayes DJ , Harmey JH . Antimetastatic activity of a cyclooxygenase‐2 inhibitor. Br J Cancer. 2004;91(2):359–365. 10.1038/sj.bjc.6601967 15213717 PMC2409822

[30] Lucotti S , Cerutti C , Soyer M , Gil‐Bernabé AM , Gomes AL , Allen PD , et al. Aspirin blocks formation of metastatic intravascular niches by inhibiting platelet‐derived COX‐1/thromboxane A2. J Clin Investig. 2019;129(5):1845–1862. 10.1172/JCI121985 30907747 PMC6486338

[31] Xu L , Stevens J , Hilton MB , Seaman S , Conrads TP , Veenstra TD , et al. COX‐2 inhibition potentiates antiangiogenic cancer therapy and prevents metastasis in preclinical models. Sci Transl Med. 2014;6(242):242ra84. 10.1126/scitranslmed.3008455 PMC6309995 24964992

[32] Ramos‐Inza S , Ruberte AC , Sanmartín C , Sharma AK , Plano D . NSAIDs: old acquaintance in the pipeline for cancer treatment and prevention‐structural modulation, mechanisms of action, and bright future. J Med Chem. 2021;64(22):16380–16421. 10.1021/acs.jmedchem.1c01460 34784195

[33] Connolly EM , Harmey JH , O'Grady T , Foley D , Roche‐Nagle G , Kay E , et al. Cyclo‐oxygenase inhibition reduces tumour growth and metastasis in an orthotopic model of breast cancer. Br J Cancer. 2002;87(2):231–237. 10.1038/sj.bjc.6600462 12107848 PMC2376100

[34] Bindu S , Mazumder S , Bandyopadhyay U . Non‐steroidal anti‐inflammatory drugs (NSAIDs) and organ damage: a current perspective. Biochem Pharmacol. 2020;180:114147. 10.1016/j.bcp.2020.114147 32653589 PMC7347500

[35] Danics L , Schvarcz CA , Viana P , Vancsik T , Krenács T , Benyó Z , et al. Exhaustion of protective heat shock response induces significant tumor damage by apoptosis after modulated electro‐hyperthermia treatment of triple negative breast cancer isografts in mice. Cancers. 2020;12(9):2581. 10.3390/cancers12092581 32927720 PMC7565562

[36] Thyagarajan A , Saylae J , Sahu RP . Acetylsalicylic acid inhibits the growth of melanoma tumors via SOX2‐dependent‐PAF‐R‐independent signaling pathway. Oncotarget. 2017;8(30):49959–49972. 28636992 10.18632/oncotarget.18326 PMC5564820

[37] Huang DW , Sherman BT , Tan Q , Kir J , Liu D , Bryant D , et al. DAVID bioinformatics resources: expanded annotation database and novel algorithms to better extract biology from large gene lists. Nucleic Acids Res. 2007;35:W169–W175. 10.1093/nar/gkm415 17576678 PMC1933169

[38] Esquivel‐Velázquez M , Ostoa‐Saloma P , Palacios‐Arreola MI , Nava‐Castro KE , Castro JI , Morales‐Montor J . The role of cytokines in breast cancer development and progression. J Interferon Cytokine Res. 2015;35(1):1–16. 10.1089/jir.2014.0026 25068787 PMC4291218

[39] Dossus L , Kaaks R , Canzian F , Albanes D , Berndt SI , Boeing H , et al. PTGS2 and IL6 genetic variation and risk of breast and prostate cancer: results from the Breast and Prostate Cancer Cohort Consortium (BPC3). Carcinogenesis. 2010;31(3):455–461. 10.1093/carcin/bgp307 19965896 PMC2832545

[40] Zappavigna S , Cossu AM , Grimaldi A , Bocchetti M , Ferraro GA , Nicoletti GF , et al. Anti‐inflammatory drugs as anticancer agents. Int J Mol Sci. 2020;21(7):2605. 10.3390/ijms21072605 32283655 PMC7177823

[41] Neeb L , Hellen P , Boehnke C , Hoffmann J , Schuh‐Hofer S , Dirnagl U , et al. IL‐1β stimulates COX‐2 dependent PGE2 synthesis and CGRP release in rat trigeminal ganglia cells. PLoS One. 2011;6(3):e17360. 10.1371/journal.pone.0017360 21394197 PMC3048859

[42] Crofford LJ , Wilder RL , Ristimäki AP , Sano H , Remmers EF , Epps HR , et al. Cyclooxygenase‐1 and ‐2 expression in rheumatoid synovial tissues effects of interleukin‐1f, phorbol ester, and corticosteroids. J Clin Invest. 1994;93(3):1095–1101. 8132748 10.1172/JCI117060 PMC294048

[43] Sorli CH , Zhang H‐J , Armstrong MB , Rajotte RV , Maclouf J , Robertson RP . Basal expression of cyclooxygenase‐2 and nuclear factor‐interleukin 6 are dominant and coordinately regulated by interleukin 1 in the pancreatic islet. Proc Natl Acad Sci U S A. 1998;95(4):1788–1793. 9465095 10.1073/pnas.95.4.1788 PMC19191

[44] Waldner MJ , Foersch S , Neurath MF . Interleukin‐6 – a key regulator of colorectal cancer development. Int J Biol Sci. 2012;8(9):1248–1253. 10.7150/ijbs.4614 23136553 PMC3491448

[45] Bell CR , Pelly VS , Moeini A , Chiang SC , Flanagan E , Bromley CP , et al. Chemotherapy‐induced COX‐2 upregulation by cancer cells defines their inflammatory properties and limits the efficacy of chemoimmunotherapy combinations. Nat Commun. 2022;13(1):2063. 10.1038/s41467-022-29606-9 35440553 PMC9018752

[46] Ferrario A , Fisher AM , Rucker N , Gomer CJ . Celecoxib and NS‐398 enhance photodynamic therapy by increasing in vitro apoptosis and decreasing in vivo inflammatory and angiogenic factors. Cancer Res. 2005;65(20):9473–9478. 10.1158/0008-5472.CAN-05-1659 16230411

[47] Mokhtari RB , Homayouni TS , Baluch N , Morgatskaya E , Kumar S , Das B , et al. Combination therapy in combating cancer. Oncotarget. 2017;8(23):38022–38043. 28410237 10.18632/oncotarget.16723 PMC5514969

[48] NHS England . Widespread clinical support for reforming NHS cancer standards to speed up diagnosis for patients.

[49] Kuo IM , Lee JJ , Wang YS , Chiang HC , Huang CC , Hsieh PJ , et al. Potential enhancement of host immunity and anti‐tumor efficacy of nanoscale curcumin and resveratrol in colorectal cancers by modulated electro‐ hyperthermia. BMC Cancer. 2020;20(1):603. 10.1186/s12885-020-07072-0 32600429 PMC7324975

[50] Mak IW , Evaniew N , Ghert M . Lost in translation: animal models and clinical trials in cancer treatment. Am J Transl Res. 2014;6(2):114–118. 24489990 PMC3902221

[51] Nair A , Jacob S . A simple practice guide for dose conversion between animals and human. J Basic Clin Pharm. 2016;7(2):27–31. 10.4103/0976-0105.177703 27057123 PMC4804402

[52] Fanaroff AC , Roe MT . Contemporary reflections on the safety of long‐term aspirin treatment for the secondary prevention of cardiovascular disease. Drug Saf. 2016;39(8):715–727. 10.1007/s40264-016-0421-1 27028617 PMC5778440

[53] Shin S . Safety of celecoxib versus traditional nonsteroidal anti‐inflammatory drugs in older patients with arthritis. J Pain Res. 2018;11:3211–3219. 10.2147/JPR.S186000 30588073 PMC6299466

[54] Xie B , Stammes MA , van Driel PB , Cruz LJ , Knol‐Blankevoort VT , Löwik MA , et al. Necrosis avid near infrared fluorescent cyanines for imaging cell death and their use to monitor therapeutic efficacy in mouse tumor models. Oncotarget. 2015;6(36):39036–39049. 26472022 10.18632/oncotarget.5498 PMC4770755

[55] Wolf BB , Schuler M , Echeverri F , Green DR . Caspase‐3 is the primary activator of apoptotic DNA fragmentation via DNA fragmentation factor‐45/inhibitor of caspase‐activated DNase inactivation. J Biol Chem. 1999;274(43):30651–30656. 10.1074/jbc.274.43.30651 10521451

[56] Pfeffer CM , Singh ATK . Apoptosis: a target for anticancer therapy. Int J Mol Sci. 2018;19(2):448. 10.3390/ijms19020448 29393886 PMC5855670

[57] Carneiro BA , El‐Deiry WS . Targeting apoptosis in cancer therapy. Nat Rev Clin Oncol. 2020;17(7):395–417. 10.1038/s41571-020-0341-y 32203277 PMC8211386

[58] Min Zhou X , Wong BC , Fan XM , Zhang HB , Lin MC , Kung HF , et al. Non‐steroidal anti‐inflammatory drugs induce apoptosis in gastric cancer cells through up‐regulation of bax and bak. Carcinogenesis. 2001;22(9):1393–1397. 11532860 10.1093/carcin/22.9.1393

[59] Lu P , Takai K , Weaver VM , Werb Z . Extracellular matrix degradation and remodeling in development and disease. Cold Spring Harb Perspect Biol. 2011;3(12):a005058. 10.1101/cshperspect.a005058 21917992 PMC3225943

[60] Cal S , López‐Otín C . ADAMTS proteases and cancer. Matrix Biol. 2015;44–46:77–85. 10.1016/j.matbio.2015.01.013 25636539

[61] Bonnans C , Chou J , Werb Z . Remodelling the extracellular matrix in development and disease. Nat Rev Mol Cell Biol. 2014;15(12):786–801. 10.1038/nrm3904 25415508 PMC4316204

[62] Kelwick R , Wagstaff L , Decock J , Roghi C , Cooley LS , Robinson SD , et al. Metalloproteinase‐dependent and ‐independent processes contribute to inhibition of breast cancer cell migration, angiogenesis and liver metastasis by a disintegrin and metalloproteinase with thrombospondin motifs‐15. Int J Cancer. 2015;136(4):E14–E26. 10.1002/ijc.29129 25099234

[63] Binder MJ , McCoombe S , Williams ED , McCulloch DR , Ward AC . ADAMTS‐15 has a tumor suppressor role in prostate cancer. Biomolecules. 2020;10(5):682. 10.3390/biom10050682 32354091 PMC7277637

[64] Porter S , Span PN , Sweep FCGJ , Tjan‐Heijnen VCG , Pennington CJ , Pedersen TX , et al. ADAMTS8 and ADAMTS15 expression predicts survival in human breast carcinoma. Int J Cancer. 2006;118(5):1241–1247. 10.1002/ijc.21476 16152618

[65] Liang L , Zhu JH , Chen G , Qin XG , Chen JQ . Prognostic values for the mRNA expression of the ADAMTS family of genes in gastric cancer. J Oncol. 2020;2020:9431560. 10.1155/2020/9431560 32884571 PMC7455834

[66] Cangemi C , Hansen ML , Argraves WS , Rasmussen LM . Fibulins and their role in cardiovascular biology and disease. Adv Clin Chem. 2014;67:245–265. 10.1016/BS.ACC.2014.09.008 25735864

[67] Zhu XJ , Liu J , Xu XY , Zhang CD , Dai DQ . Novel tumor‐suppressor gene epidermal growth factor‐containing fibulin‐like extracellular matrix protein 1 is epigenetically silenced and associated with invasion and metastasis in human gastric cancer. Mol Med Rep. 2014;9(6):2283–2292. 10.3892/mmr.2014.2135 24718752

[68] Kaur J , Reinhardt DP . Extracellular matrix (ECM) molecules. In: Vishwakarma A , Sharpe P , Shi S , Ramalingam M , editors. Stem cell biology and tissue engineering in dental sciences. Academic Press; 2015. p. 25–45. 10.1016/B978-0-12-397157-9.00003-5

[69] Sadr‐Nabavi A , Ramser J , Volkmann J , Naehrig J , Wiesmann F , Betz B , et al. Decreased expression of angiogenesis antagonist EFEMP1 in sporadic breast cancer is caused by aberrant promoter methylation and points to an impact of EFEMP1 as molecular biomarker. Int J Cancer. 2009;124(7):1727–1735. 10.1002/ijc.24108 19115204

[70] Albig AR , Neil JR , Schiemann WP . Fibulins 3 and 5 antagonize tumor angiogenesis in vivo. Cancer Res. 2006;66(5):2621–2629. 10.1158/0008-5472.CAN-04-4096 16510581

[71] Fararjeh AS , Kaddumi E , Al Khader A , Aloliqi AA . The diagnostic and prognostic significance of EFEMP1 in breast cancer: an immunohistochemistry study. Int J Surg Pathol. 2023;31(6):1057–1066. 10.1177/10668969221126122 36259327

[72] Zhang X , Wu J , Hu C , Zheng X , Guo Z , Li L . CXCL11 negatively regulated by MED19 favours antitumour immune infiltration in breast cancer. Cytokine. 2023;162:156106. 10.1016/j.cyto.2022.156106 36512935

[73] Li F , Zhang H , Wang Y , Yao Z , Xie K , Mo Q , et al. FGFBP1 as a potential biomarker predicting bacillus Calmette‐Guérin response in bladder cancer. Front Immunol. 2022;13:954836. 10.3389/fimmu.2022.954836 36119059 PMC9478507

[74] Czubayko F , Liaudet‐Coopman EDE , Aigner A , Tuveson AT , Berchem GJ , Wellsteln A . A secreted FGF‐binding protein can serve as the angiogenic switch in human cancer. Nat Med. 1997;3(10):1137–1140. 9334727 10.1038/nm1097-1137

[75] Chang ACM , Doherty J , Huschtscha LI , Redvers R , Restall C , Reddel RR , et al. STC1 expression is associated with tumor growth and metastasis in breast cancer. Clin Exp Metastasis. 2015;32(1):15–27. 10.1007/s10585-014-9687-9 25391215

[76] Chen F , Zhang Z , Pu F . Role of stanniocalcin‐1 in breast cancer. Oncol Lett. 2019;18(4):3946–3953. 10.3892/ol.2019.10777 31579413 PMC6757304

[77] Pemmari A , Tuure L , Hämäläinen M , Leppänen T , Moilanen T , Moilanen E . Effects of ibuprofen on gene expression in chondrocytes from patients with osteoarthritis as determined by RNA‐Seq. RMD Open. 2021;7(3):e001657. 10.1136/rmdopen-2021-001657 34497153 PMC8438934

[78] Magnussen SN , Toraskar J , Wilhelm I , Hasko J , Figenschau SL , Molnar J , et al. Nephronectin promotes breast cancer brain metastatic colonization via its integrin‐binding domains. Sci Rep. 2020;10(1):12237. 10.1038/s41598-020-69242-1 32699247 PMC7376038

[79] van Kooyk Y , Ilarregui JM , van Vliet SJ . Novel insights into the immunomodulatory role of the dendritic cell and macrophage‐expressed C‐type lectin MGL. Immunobiology. 2015;220(2):185–192. 10.1016/j.imbio.2014.10.002 25454488

[80] Shaoqing LIU , Zhang J , Jiujun ZHU , Jiao D , Zhenzhen LIU . Prognostic values of EDNRB in triple‐negative breast cancer. Oncol Lett. 2020;20(5):149. 10.3892/OL.2020.12012 32934717 PMC7471672

[81] Jenkins BD , Martini RN , Hire R , Brown A , Bennett B , Brown I' , et al. Atypical chemokine receptor 1 (DARC/ACKR1) in breast tumors is associated with survival, circulating chemokines, tumor‐infiltrating immune cells, and African ancestry. Cancer Epidemiol Biomarkers Prev. 2019;28(4):690–700. 10.1158/1055-9965.EPI-18-0955 30944146 PMC6450416

[82] Peppino G , Ruiu R , Arigoni M , Riccardo F , Iacoviello A , Barutello G , et al. Teneurins: role in cancer and potential role as diagnostic biomarkers and targets for therapy. Int J Mol Sci. 2021;22(5):2321. 10.3390/ijms22052321 33652578 PMC7956758

[83] Zhang R , Jiang HX , Liu YJ , He GQ . Structure, function, and pathology of neurexin‐3. Genes Dis. 2022;10(5):1908–1919. 10.1016/j.gendis.2022.04.008 37492720 PMC10363586

[84] Hanahan D , Monje M . Cancer hallmarks intersect with neuroscience in the tumor microenvironment. Cancer Cell. 2023;41(3):573–580. 10.1016/j.ccell.2023.02.012 36917953 PMC10202656

[85] Dai D , Liu H . The nervous system contributes to the tumorigenesis and progression of human digestive tract cancer. J Immunol Res. 2022;2022:9595704. 10.1155/2022/9595704 35295188 PMC8920690

[86] Kusinska R , Górniak P , Pastorczak A , Fendler W , Potemski P , Mlynarski W , et al. Influence of genomic variation in FTO at 16q12.2, MC4R at 18q22 and NRXN3 at 14q31 genes on breast cancer risk. Mol Biol Rep. 2012;39(3):2915–2919. 10.1007/s11033-011-1053-2 21688152 PMC3271204

[87] Fox BP , Kandpal RP . EphB6 receptor significantly alters invasiveness and other phenotypic characteristics of human breast carcinoma cells. Oncogene. 2009;28(14):1706–1713. 10.1038/onc.2009.18 19234485

[88] Osta WA , Chen Y , Mikhitarian K , Mitas M , Salem M , Hannun YA , et al. EpCAM is overexpressed in breast cancer and is a potential target for breast cancer gene therapy. Cancer Res. 2004;64(16):5818–5824. 15313925 10.1158/0008-5472.CAN-04-0754

[89] Gao S , Sun Y , Liu X , Zhang D , Yang X . EpCAM and COX‐2 expression are positively correlated in human breast cancer. Mol Med Rep. 2017;15(6):3755–3760. 10.3892/mmr.2017.6447 28393249

